# Efficient Synthesis of Readily Water-Soluble Sulfonic Acid Carbamates

**DOI:** 10.3390/molecules20046856

**Published:** 2015-04-16

**Authors:** Krzysztof R. Idzik, Karsten Nödler, Tobias Licha

**Affiliations:** Department Applied Geology, Geoscience Centre of the University of Göttingen, Goldschmidtstrasse 3, 37077 Göttingen, Germany; E-Mails: karsten.noedler@geo.uni-goettingen.de (K.N.); tobias.licha@geo.uni-goettingen.de (T.L.)

**Keywords:** chloroformates, primary amines, carbamates, thermo-sensitive tracers, urethanes

## Abstract

A series of various readily water-soluble carbamates were synthesized with good yields. These compounds are useful chemical tracers for assessing the cooling progress in a georeservoir during geothermal power plant operation. Acylation of primary amines was carried out as well as using a solution of sodium bicarbonate and without the presence of salt. Products were characterized by ^1^H-NMR and ^13^C-NMR. Purity was confirmed through elemental analysis.

## 1. Introduction

Carbamates play an important role in medicine as biologically active compounds; for example, quer-Cetin-carbamate and Tricine-carbamate are prodrugs [1,2,3]. These compounds are efficiently transported by the oligopeptide transporter 1 (PepT1), a membrane transport protein localized in the brush-border membranes of intestinal epithelial cells, resulting in significantly enhanced oral bioavailability of the parent drugs [4]. Moreover, studies have revealed that in these compounds, l-amino acid carbamate structural fragments are crucial for enhanced water solubility and cell permeability [5].

Carbamates are also known as urethanes, e.g., ethyl carbamate occurs naturally in many fermented foods and beverages [6,7,8]. The precursor of ethyl carbamate is also well-known to be genetically related to lactic acid bacteria [9]. On the other hand, urethanes as polymers (polyurethanes SPUs) have been utilized in medical devices since the 1960s due to their established biocompatibility, excellent mechanical properties, and extended fatigue life [10], which allows their use in several medical device designs, including but not limited to cardiac pacemaker and neurological lead coatings [11], infusion pumps [12], dialysis catheters [13], vascular grafts [14], intervertebral discs, acetabular cups [15], and arteriovenous shunts [16], where bioresistant materials are critical. 

It is also worth mentioning that specific compounds from the class of carbamates are widely used as insecticides and fungicides in agriculture. Their mode of action is in affecting the nervous system by disrupting an enzyme that regulates acetylcholine, a neurotransmitter [17]. These compounds are considered hazardous to the environment and human health. They are on the priority list published by the US Environmental Protection Agency (EPA) [18,19].

As demonstrated, carbamates are used in many different applications. We are interested in this class of compounds because they are potentially useful to investigate the temperature distribution of deep geological formations. Currently, there is an increasing demand for reactive tracers with thermo-sensitive properties for improved reservoir management in geothermal systems with thermal drawdown. Promising tracer candidates are compounds susceptible to undergoing hydrolysis, which are of greater interest for geothermal applications [20,21]. This is mainly caused by the fact that the reservoir temperature is not directly accessible. To estimate the thermal state of a georeservoir, two slightly different thermo-sensitive tracer approaches are currently used. One uses the non-specific thermal decay kinetics of established tracers (e.g., naphthalene sulfonates, fluorescein) at high temperatures while the other approach exploits the structure-related kinetics of defined hydrolysis reactions (e.g., phenol acetates). Up to now, though, successful tracer application has been limited to certain geothermal temperature conditions due to either the non-availability of suitable purchasable substances or the lack of knowledge on structure-dependent kinetics. To fill these gaps and to improve the general applicability of thermo-sensitive tracers, a wide spectrum of hydrolyzable compounds with thermo-sensitive properties was synthesized in our working group. The concept of structure-dependent kinetics learnt from phenolic esters [20] was transferred to carbamates. Their thermally induced hydrolysis leads to well known reaction products with fluorescent properties in a first-order reaction. These novel carbamates with sulfonic groups and thus a desired high water solubility (>1 kg/L) differ in their substructures and thus should also differ in their reaction kinetics. 

Carbamates are generally obtained from an amine by the reaction with an adequate chloroformate or anhydride. Some of the known methods involve carbonylation of amine or nitro compounds, oxidative carbonylation of amines, the carboxylation reaction between amines and carbon dioxide, alcoholysis of urea, and carboxylation of amines involving carbonate as carbonylat. Carbamates are conventionally synthesized on industrial scale by the phosgene route [22]. Carbamates are also obtained by combining isocyanates, e.g., toluene isocyanate with compounds containing hydroxyl groups, where the carbamate groups are formed by the reaction of the alcohols or phenols with the isocyanates. This reaction can be catalyzed by aluminum chloride [23,24,25]. The isocyanates used in these reactions are obtained by phosgenation of the corresponding amines or the corresponding alcohols. The drawback of these processes is that they are very expensive. Furthermore, phosgene has to be used with care because of its human toxicity and ecotoxicity. 

Carbamates may also be produced by the reaction of an amine with carbonate in presence of a catalytic quantity of a Lewis acid based on zinc or tin compounds. The drawback of this process is that alcohols are always produced as byproducts and these need to be removed during the purification of the carbamates [26]. Franz *et al.* described a process for making methyl-*N*-phenyl carbamate from carbon monoxide, sulphur, aniline, and methanol. Very low yields are produced by this method; the yield did not exceed 25% even over extended reaction periods [27]. 

*N*-arylcarbamates can be prepared by letting nitroaromatics react with carbon monoxide and alcohols in the presence of catalysts [28]. Thus, carbamates may be prepared by the reaction of organic nitro compounds, carbon monoxide, and hydroxyl-containing compounds in the presence of a catalysts consisting of a noble metal and a Lewis acid under essentially anhydrous conditions in the absence of hydrogen under increased pressure and at temperatures above 150 °C.

Acylation provides an inexpensive and efficient way for obtaining carbamates through a synthetic process. In this paper we present an efficient synthesis of readily water-soluble carbamates containing sulfonic groups obtained from the reaction of amines with chloroformates.

## 2. Results and Discussion

Our goal was to develop a simple method for the preparation of carbamates that begins with readily available starting materials and can be carried out under mild reaction conditions while being highly efficient and economical. For our initial studies, we selected readily available methyl chloroformate, propyl chloroformate, phenyl, tolyl, and naphthalyl chloroformate as the “carbonyl source” to prepare carbamates from corresponding amines. Although the preparation of corresponding carbamates was straightforward, finding a suitable method for the preparation and purification of water-soluble carbamates containing sulfonic groups was not trivial. 

**Scheme 1:**
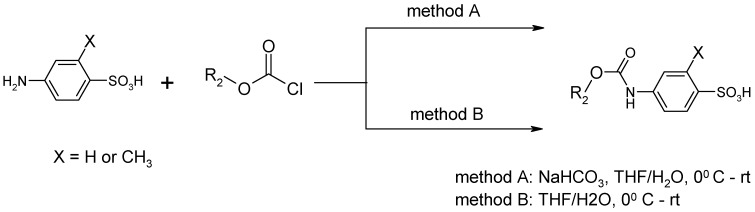
Synthesis carbamates (**A**) with sodium bicarbonate; (**B**) without any bases.

Our procedure utilizes readily available aromatic and aliphatic chloroformates and the corresponding amines as reactants in THF/water media with sodium bicarbonate as a base (Scheme 1A). Reaction was also performed without any base (Scheme 1B). The reaction is carried out at 0 °C to prevent phenyl carbamate decomposition and is complete after all reagents are added. 

The mixture was stirred at room temperature for two days. The product was isolated by solvent evaporation, and purified by recrystallization from methanol-water solution or purified by column chromatography. Yields are presented in Table 1.

**Table 1:** Carbamates.

Entry	Substrate 1 (R_1_-NH_2_)	Substrate 2 (R_2_-OCOCl)	Product (R_1_-NHOCO-R_2_)	Yield (%)		
				A	B
1	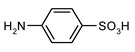	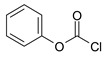	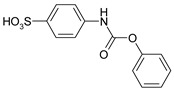	99	99
2	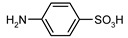	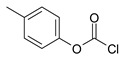	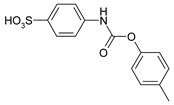	98	98
3	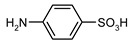	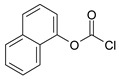	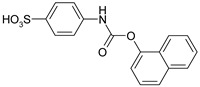	98	97
4	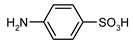	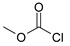	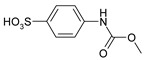	99	98
5	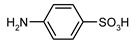	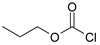	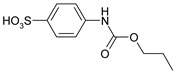	98	96
6	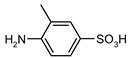	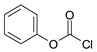	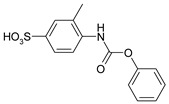	98	98
7	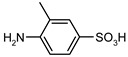	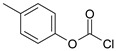	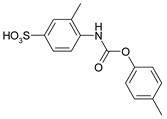	97	94
8	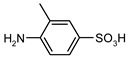	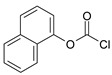	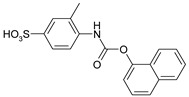	99	95
9	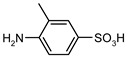	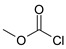	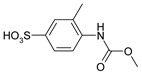	97	97
10	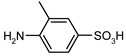	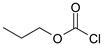	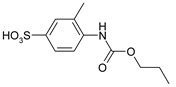	97	96

The solid carbamates are stable at room temperature for at least several months, making them an excellent intermediate. To determine optimal reaction conditions for the preparation of carbamates we performed experiments with varying solvents, temperatures, and reaction times. The best results are received in the method described below. Acylation of primary amines is carried out in water-THF solution using a stoichiometric amount of chloroformate followed by addition of saturated aqueous bicarbonate solution. The primary amine is dissolved in water and then an equivalent amount of chloroformate derivative in THF (1:1) is added dropwise under stirring. Primary amines containing protonated sulfonic groups are very poorly soluble in water and in this case must be previously heated till completely dissolved in water and after that slowly cooled to room temperature. The desired product is finally obtained by treating the reaction mixture with saturated bicarbonate solution. The products are crystallized from methanol/water solution or purified by column chromatography.

The main problem in using method A for water soluble compounds is the separation of resulting products from salts in the purification step. We tried to find an alternative method for obtaining water soluble carbamates without the presence of bicarbonate solution. It should be noted that the reaction also works very well without the presence of sodium bicarbonate. The yields of both methods are comparable. We think that the presence of sodium bicarbonate only slightly improves the yield of the reaction. Comparing the results of the two methods, we believed that the use of salt is unnecessary and only increases the cost of carbamate production. Highest yields were obtained at room temperature after reaction times of 2 days. By applying method B it is possible to obtain products free of inorganic salts. The results of both methods are summarized in Table 1.

According to our previously experience in the preparation of amides [29] and esters [30], we tried to use this method to obtain carbamates. A single-phase solvent system consisting of ethyl acetate as an organic solvent and triethylamine as a catalyst was used. Unfortunately, we did not observe any product formation.

We have developed an exceptionally simple, high yield, and economical procedure for the preparation of substituted carbamates from readily available amines and chloroformates. This condensation reaction can even be performed with aromatic and aliphatic groups. Not only is this approach applicable to large scale industrial syntheses, it is also applicable to the synthesis of carbamates under laboratory conditions.

## 3. Experimental Section

### 3.1. General

All chemicals, reagents, and solvents were used as received from commercial sources without further purification. ^1^H-NMR and ^13^C-NMR spectra were recorded in DMSO-*d_6_* on a 300 MHz liquid state Bruker spectrometer. The splitting patterns are annotated as follows: s (singlet), d (doublet), t (triplet), q (quartet) and m (multiplet). The products were also confirmed by ESI-MS (instrumentation is described in Nödler *et al.* [31]). Preparative column chromatography was carried out on glass columns of different sizes packed with silica gel: Merck 60 (0.035–0.070 mm).

### 3.2. Synthesis

Method A. General procedure of preparing carbamates containing sulfonic groups (in presence of sodium bicarbonate) **1**–**10**. A solution of chloroformate (2.0 mmol) in tetrahydrofuran (25 mL) was slowly added to an ice-water bath cooled solution of primary amine (2.0 mmol) (primary amines containing sulfonic groups are very poorly soluble in cold water and must previously be heated until completely dissolved in water and after that cooled to rt.) and water solution of sodium bicarbonate (3.0 mmol). After the addition was complete, the ice-water bath was removed and the reaction mixture left to stir at room temperature for two days. After that, aqueous hydrochloric acid was added and the mixture was stirring for 2 h. The solvent was evaporated to give the crude product, which was subsequently purified using column chromatography (hexane/ethyl acetate, 1:5 and then methanol).

Method B. General procedure of preparing carbamates containing sulfonic groups (without presence of sodium bicarbonate) **1**–**10**. A solution of chloroformate (2.0 mmol) in tetrahydrofuran (30 mL) was slowly added to an ice-water bath cooled solution of primary amine (2.0 mmol) in water (30 mL) (primary amines containing sulfonic groups are very poorly soluble in cold water and must be previously heated till completely dissolve in water and after that cooled to rt.). After the addition was complete, the ice-water bath was removed and the reaction mixture left to stir at room temperature for two days. The solvent was evaporated to give the crude product, which was subsequently purified using column chromatography (hexane/ethyl acetate, 1:5 and then methanol).

*4-[(Phenoxycarbonyl)amino]benzenesulfonic acid* (**1**) Yield: 0.58 g (99%). White powder. ^1^H-NMR (300 MHz, DMSO-*d_6_*) δ, ppm, 10.23 (s, 1H), 7.57 (d, *J* = 8.1 Hz, 2H), 7.44 (d, *J* = 7.8 Hz, 2H), 7.39 (d, *J* = 7.8 Hz, 2H), 7.24 (t, *J* = 7.4 Hz, 1H), 7.18 (d, *J* = 7.8 Hz, 2H); ^13^C-NMR (300 MHz, DMSO-*d_6_*) δ, ppm, 151.93, 150.52, 142.48, 139.05, 129.58, 126.48, 125.65, 121.96, 117.76. Elemental analysis for: C_13_H_11_NO_5_S Calc.: C, 53.24; H, 3.78. Found: C, 53.32; H, 3.67. ESI-MS: *m/z* = 293.

*4-{[(4-Methylphenoxy)carbonyl]amino}benzenesulfonic acid* (**2**) Yield: 0.60 g (98%). White powder. ^1^H-NMR (300 MHz, DMSO-*d_6_*) δ, ppm, 10.19 (s, 1H), 7.55 (d, *J* = 8.5 Hz, 2H), 7.44 (d, *J* = 8.5 Hz, 2H), 7.20 (d, *J* = 8.5 Hz, 2H), 7.09 (d, *J* = 8.5 Hz, 2H), 2.31 (s, 3H); ^13^C-NMR (300 MHz, DMSO-*d_6_*) δ, ppm, 151.54, 148.02, 142.79, 138.49, 134.29, 129.49, 126.07, 121.38, 117.15, 20.30. Elemental analysis for: C_14_H_13_NO_5_S Calc.: C, 54.72; H, 4.26. Found: C, 54.82; H, 4.36. ESI-MS: *m/z* = 307.

*4-{[(Naphthalen-1-yloxy)carbonyl]amino}benzenesulfonic acid* (**3**) Yield: 0.67 g (97%). Light brown powder. ^1^H-NMR (300 MHz, DMSO-*d_6_*) δ, ppm, 10.53 (s, 1H), 8.08–7.96 (m, 2H), 7.86 (d, *J* = 8.0 Hz, 2H), 7.69–7.49 (m, 5H), 7.43 (d, *J* = 7.6 Hz, 2H); ^13^C-NMR (300 MHz, DMSO-*d_6_*) δ, ppm, 151.59, 145.96, 143.05, 138.53, 133.99, 127.80, 126.88, 126.51, 126.39, 126.20, 125.59, 125.41, 120.84, 118.51, 117.31. Elemental analysis for: C_17_H_13_NO_5_S Calc.: C, 59.47; H, 3.82. Found: C, 59.52; H, 3.76. ESI-MS: *m/z* = 343.

*4-[(Methoxycarbonyl)amino]benzenesulfonic acid* (**4**) Yield: 0.45 g (98%). Brown powder. ^1^H-NMR (300 MHz, DMSO-*d_6_*) δ, ppm, 9.67 (s, 1H), 7.52 (d, *J* = 8.5 Hz, 2H), 7.39 (d, *J* = 8.5 Hz, 2H), 3.66 (s, 3H); ^13^C-NMR (300 MHz, DMSO-*d_6_*) δ, ppm, 153.70, 142.13, 139.05, 125.99, 116.84, 51.53. Elemental analysis for: C_8_H_9_NO_5_S Calc.: C, 41.56; H, 3.92. Found: C, 41.72; H, 3.75. ESI-MS: *m/z* = 231.

*4-[(Propoxycarbonyl)amino]benzenesulfonic acid* (**5**) Yield: 0.50 g (96%). Dark brown powder. ^1^H-NMR (300 MHz, DMSO-*d_6_*) δ, ppm, 9.63 (s, 1H), 7.52 (d, *J* = 8.8 Hz, 2H), 7.41 (d, *J* = 8.4 Hz, 2H), 4.01 (t, *J* = 6.6 Hz, 2H), 1.65–1.56 (m, 2H), 0.90 (t, *J* = 7.4 Hz, 3H); ^13^C-NMR (300 MHz, DMSO-*d_6_*) δ, ppm, 153.17, 141.66, 139.08, 125.76, 116.62, 65.29, 21.42, 9.77. Elemental analysis for: C_10_H_13_NO_5_S Calc.: C, 46.32; H, 5.05. Found: C, 46.53; H, 5.17. ESI-MS: *m/z* = 259.

*3-Methyl-4-[(phenoxycarbonyl)amino]benzenesulfonic acid* (**6**) Yield: 0.60 g (98%). White powder. ^1^H-NMR (300 MHz, DMSO-*d_6_*) δ, ppm, 9.41 (s, 1H), 7.47 (s, 1H), 7.42–7.35 (m, 4H), 7.23 (t, *J* = 6.0 Hz, 1H), 7.20 (d, *J* = 6.0 Hz, 2H), 2.29 (s, 3H); ^13^C-NMR (300 MHz, DMSO-*d_6_*) δ, ppm, 152.53, 150.76, 144.87, 135.99, 130.93, 129.30, 127.65, 125.19, 124.20, 123.50, 121.75, 17.73. Elemental analysis for: C_14_H_13_NO_5_S Calc.: C, 54.72; H, 4.26. Found: C, 54.83; H, 4.18. ESI-MS: *m/z* = 307.

*3-Methyl-4-{[(4-methylphenoxy)carbonyl]amino}benzenesulfonic acid* (**7**) Yield: 0.60 g (94%). Brown powder. ^1^H-NMR (300 MHz, DMSO-*d_6_*) δ, ppm, 9.37 (s, 1H), 7.48 (s, 1H), 7.43 (d, *J* = 8.4 Hz, 1H), 7.37 (d, *J* = 8.4 Hz, 1H), 7.21 (d, *J* = 8.4 Hz, 2H), 7.08 (d, *J* = 8.4 Hz, 2H), 2.31 (s, 3H), 2.28 (s, 3H); ^13^C-NMR (300 MHz, DMSO-*d_6_*) δ, ppm, 152.62, 148.50, 144.96, 135.92, 134.24, 130.76, 129.58, 127.58, 123.56, 123.43, 121.43, 20.26, 17.69. Elemental analysis for: C_15_H_15_NO_5_S Calc.: C, 56.06; H, 4.70. Found: C, 55.87; H, 4.58. ESI-MS: *m/z* = 321.

*3-Methyl-4-{[(naphthalen-1-yloxy)carbonyl]amino}benzenesulfonic acid* (**8**) Yield: 0.68 g (95%). Brown powder. ^1^H-NMR (300 MHz, DMSO-*d_6_*) δ, ppm, 9.72 (s, 1H), 8.03–7.98 (m, 2H), 7.84 (d, *J* = 8.0 Hz, 1H), 7.64–7.52 (m, 4H), 7.46–7.40 (m, 3H), 2.36 (s, 3H); ^13^C-NMR (300 MHz, DMSO-*d_6_*) δ, ppm, 152.57, 146.30, 145.14, 135.83, 134.04, 130.99, 127.83, 127.63, 126.99, 126.48, 126.40, 125.63, 125.32, 124.07, 123.50, 120.91, 118.47, 17.74. Elemental analysis for: C_18_H_15_NO_5_S Calc.: C, 60.49; H, 4.23. Found: C, 60.37; H, 4.48. ESI-MS: *m/z* = 357.

*4-[(Methoxycarbonyl)amino]-3-methylbenzenesulfonic acid* (**9**) Yield: 0.48 g (97%). Brown powder. ^1^H-NMR (300 MHz, DMSO-*d_6_*) δ, ppm, 8.80 (s, 1H), 7.42 (s, 1H), 7.38 (d, *J* = 7.5 Hz, 1H), 7.30 (d, *J* = 7.5 Hz, 1H), 3.64 (s, 3H), 2.19 (s, 3H); ^13^C-NMR (300 MHz, DMSO-*d_6_*) δ, ppm, 154.47, 144.23, 136.26, 130.17, 127.31, 123.20, 123.10, 51.56, 17.68. Elemental analysis for: C_9_H_11_NO_5_S Calc.: C, 44.08; H, 4.52. Found: C, 44.16; H, 4.69. ESI-MS: *m/z* = 245.

*3-Methyl-4-[(propoxycarbonyl)amino]benzenesulfonic acid* (**10**) Yield: 0.52 g (96%). Brown powder. ^1^H-NMR (300 MHz, DMSO-*d_6_*) δ, ppm, 8.78 (s, 1H), 7.46 (s, 1H), 7.42 (d, *J* = 8.4 Hz, 1H), 7.32 (d, *J* = 8.4 Hz, 1H), 4.01 (t, *J* = 6.6 Hz, 2H), 2.20 (s, 3H), 1.66–1.57 (m, 2H), 0.91 (t, *J* = 7.4 Hz, 3H); ^13^C-NMR (300 MHz, DMSO-*d_6_*) δ, ppm, 154.52, 143.90, 136.92, 130.63, 127.64, 124.35, 123.51, 65.85, 22.02, 17.83, 10.30. Elemental analysis for: C_11_H_15_NO_5_S Calc.: C, 48.34; H, 5.53. Found: C, 48.13; H, 5.36. ESI-MS: *m/z* = 273.

## 4. Conclusions

We have developed a very simple, inexpensive, nontoxic, and environmentally friendly method for the preparation of carbamates containing sulfonic groups. The presence of the SO_3_H group in all synthesized carbamates ensures their good water solubility as their respective salts and thus their application as tracers in geo-reservoirs. In particular, there is not much information related to the synthesis of readily water-soluble carbamates. We strongly feel that this study will find numerous applications even beyond tracers for geothermal applications. 

## Supplementary Materials

Supplementary materials can be accessed at: http://www.mdpi.com/1420-3049/20/04/6856/s1.
