# Supplementary material for: Efficient Synthesis of Readily Water-Soluble Sulfonic Acid Carbamates
Source: Molecules. 2015 Apr 16;20(4):6856–65. doi: 10.3390/molecules20046856 (PMC6272615; doi:10.3390/molecules20046856)
Supplement: Supplementary file 1 [file molecules-20-06856-s001.pdf]

Supplementary Materials

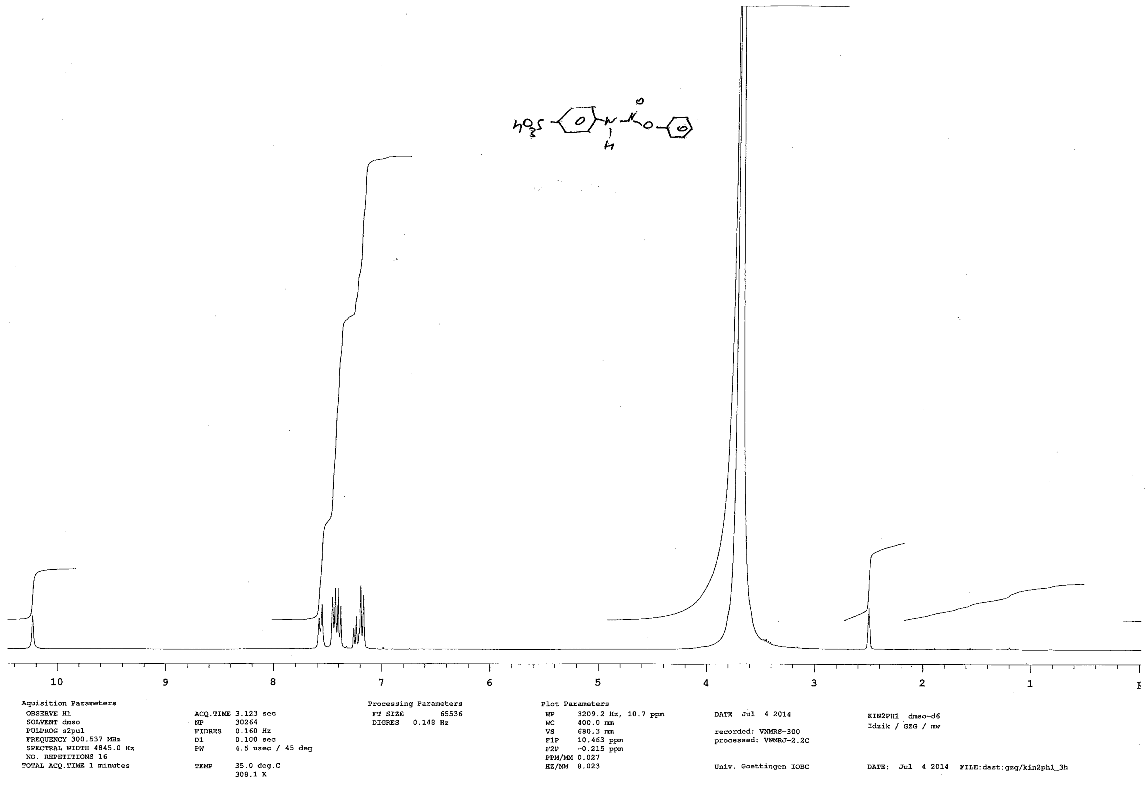

Figure S1. ^{1}H-NMR spectrum of 4-[(phenoxycarbonyl)amino]benzenesulfonic (**1**). ^{1}H-NMR (300 MHz, DMSO-*d6*) δ, ppm, 10.23 (s, 1H), 7.57 (d, *J* = 8.1 Hz, 2H), 7.44 (d, *J* = 7.8 Hz, 2H), 7.39 (d, *J* = 7.8 Hz, 2H), 7.24 (t, *J* = 7.4 Hz, 1H), 7.18 (d, *J* = 7.8 Hz, 2H).

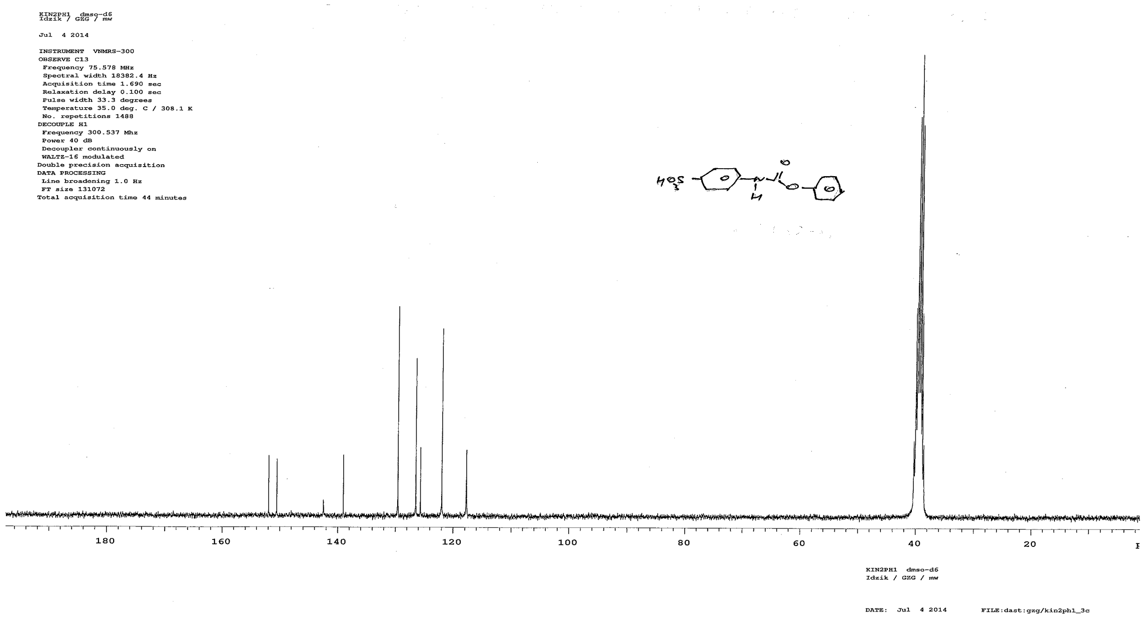

Figure S2. ^{13}C-NMR spectrum of 4-[(phenoxycarbonyl)amino]benzenesulfonic (**1**). ^{13}C-NMR (300 MHz, DMSO-*d6*) δ, ppm, 151.93, 150.52, 142.48, 139.05, 129.58, 126.48, 125.65, 121.96, 117.76.

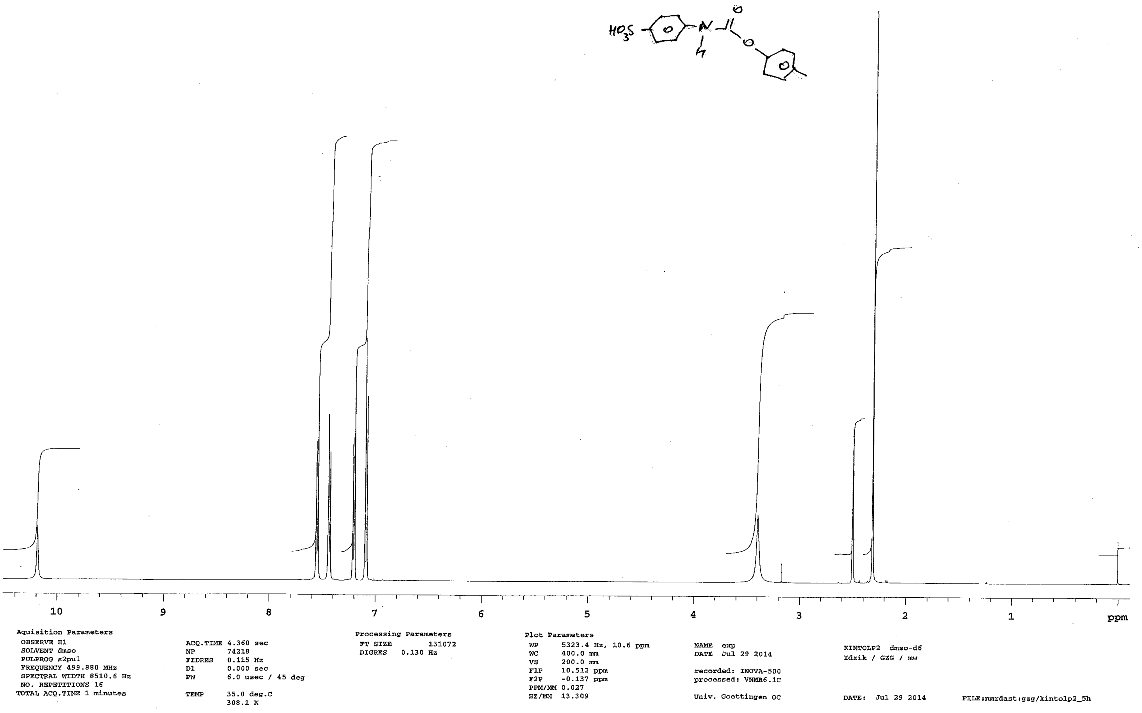

Figure S3. ^{1}H-NMR spectrum of 4-{[(4-methylphenoxy)carbonyl]amino}benzenesulfonic acid (**2**). ^{1}H-NMR (300 MHz, DMSO-d_6) δ, ppm, 10.19 (s, 1H), 7.55 (d, J = 8.5 Hz, 2H), 7.44 (d, J = 8.5 Hz, 2H), 7.20 (d, J = 8.5 Hz, 2H), 7.09 (d, J = 8.5 Hz, 2H), 2.31 (s, 3H).

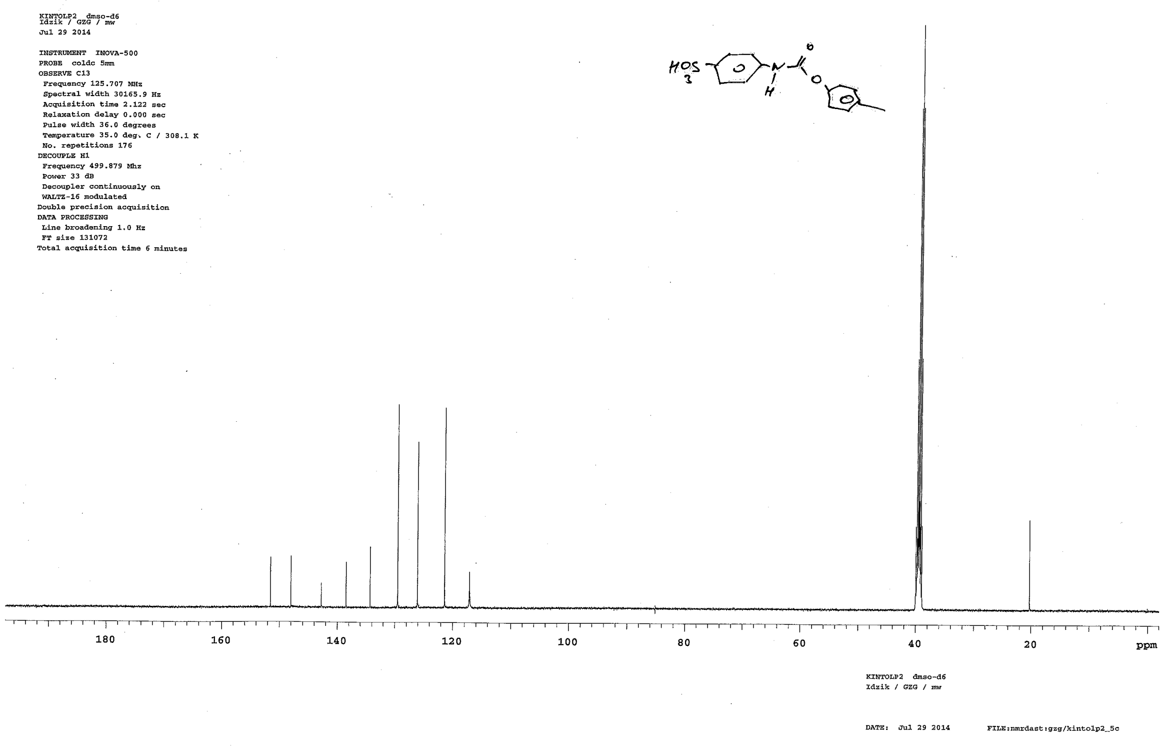

Figure S4. ^{13}C-NMR spectrum of 4-{[(4-methylphenoxy)carbonyl]amino}benzenesulfonic acid (**2**). ^{13}C-NMR (300 MHz, DMSO-d_6) δ, ppm, 151.54, 148.02, 142.79, 138.49, 134.29, 129.49, 126.07, 121.38, 117.15, 20.30.

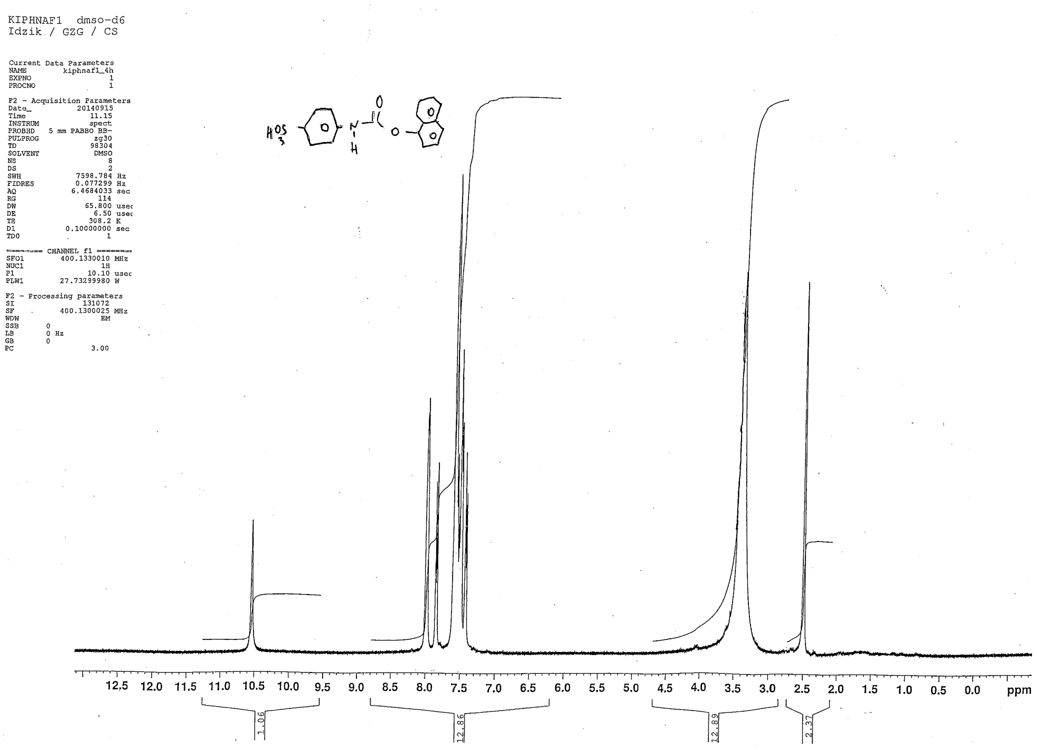

Figure S5. ^{1}H-NMR spectrum of 4-{[(naphthalen-1-yloxy)carbonyl]amino}benzenesulfonic acid (**3**). ^{1}H-NMR (300 MHz, DMSO-*d6*) δ, ppm, 10.53 (s, 1H), 8.08–7.96 (m, 2H), 7.86 (d, *J* = 8.0 Hz, 2H), 7.69–7.49 (m, 5H), 7.43 (d, *J* = 7.6 Hz, 2H).

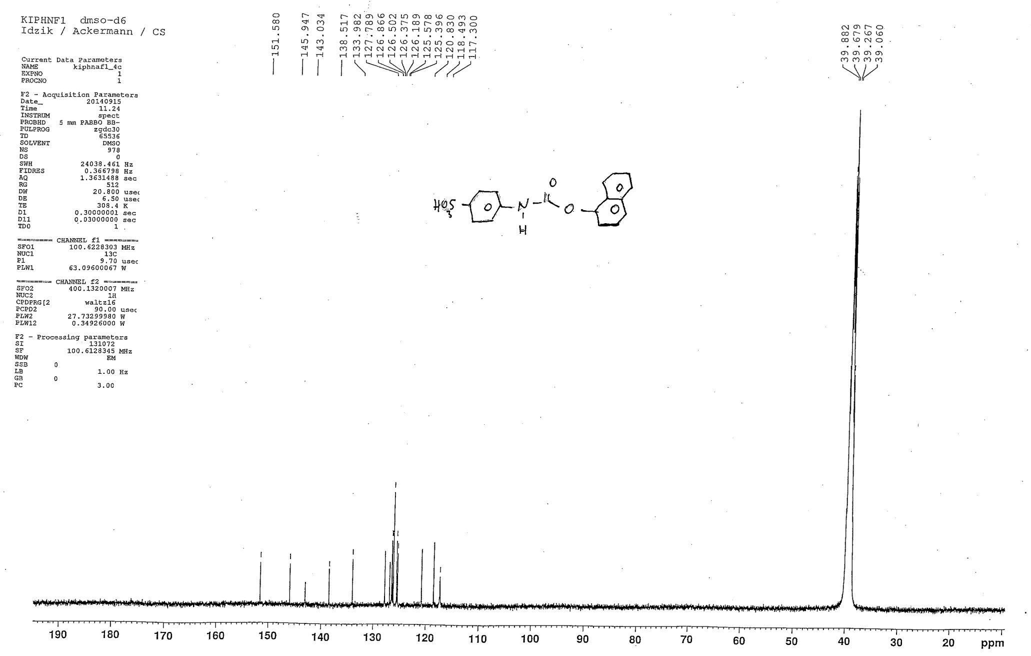

Figure S6. ^{13}C-NMR Spectrum of 4-{[(naphthalen-1-yloxy)carbonyl]amino}benzenesulfonic acid (**3**). ^{13}C-NMR (300 MHz, DMSO-*d6*) δ, ppm, 151.59, 145.96, 143.05, 138.53, 133.99, 127.80, 126.88, 126.51, 126.39, 126.20, 125.59, 125.41, 120.84, 118.51, 117.31.

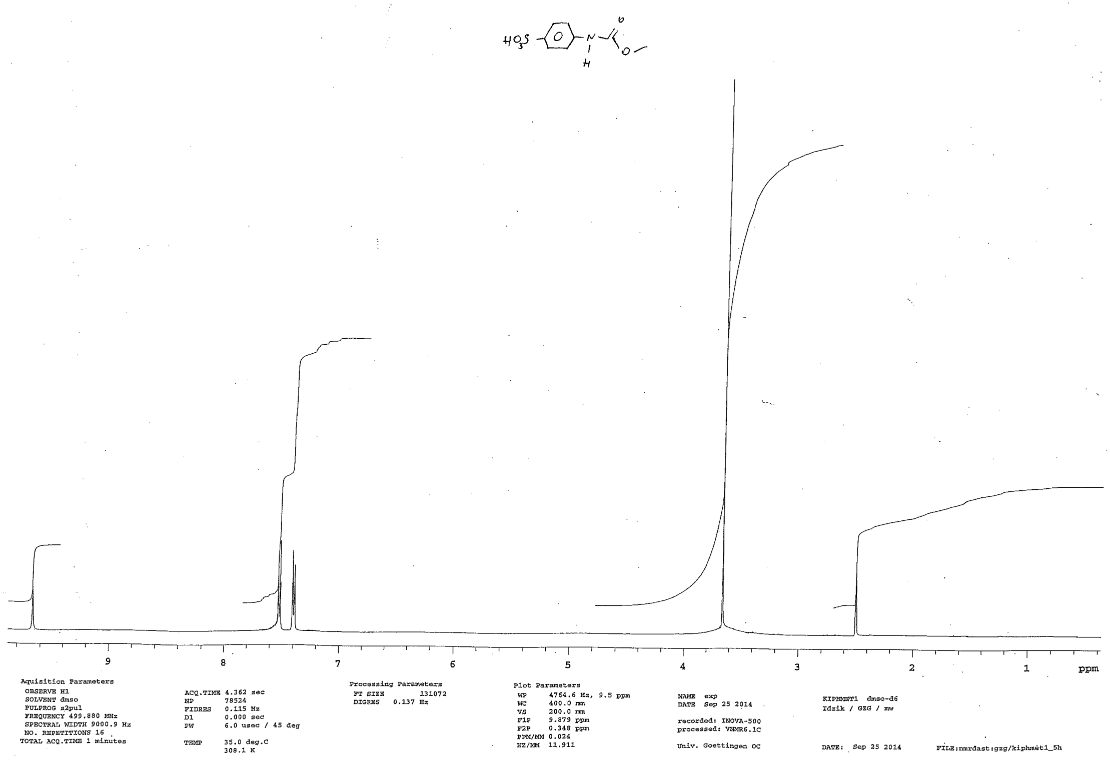

Figure S7. ^{1}H-NMR spectrum of 4-[(methoxycarbonyl)amino]benzenesulfonic acid (**4**). ^{1}H-NMR (300 MHz, DMSO-d_6) δ, ppm, 9.67 (s, 1H), 7.52 (d, J = 8.5 Hz, 2H), 7.39 (d, J = 8.5 Hz, 2H), 3.66 (s, 3H).

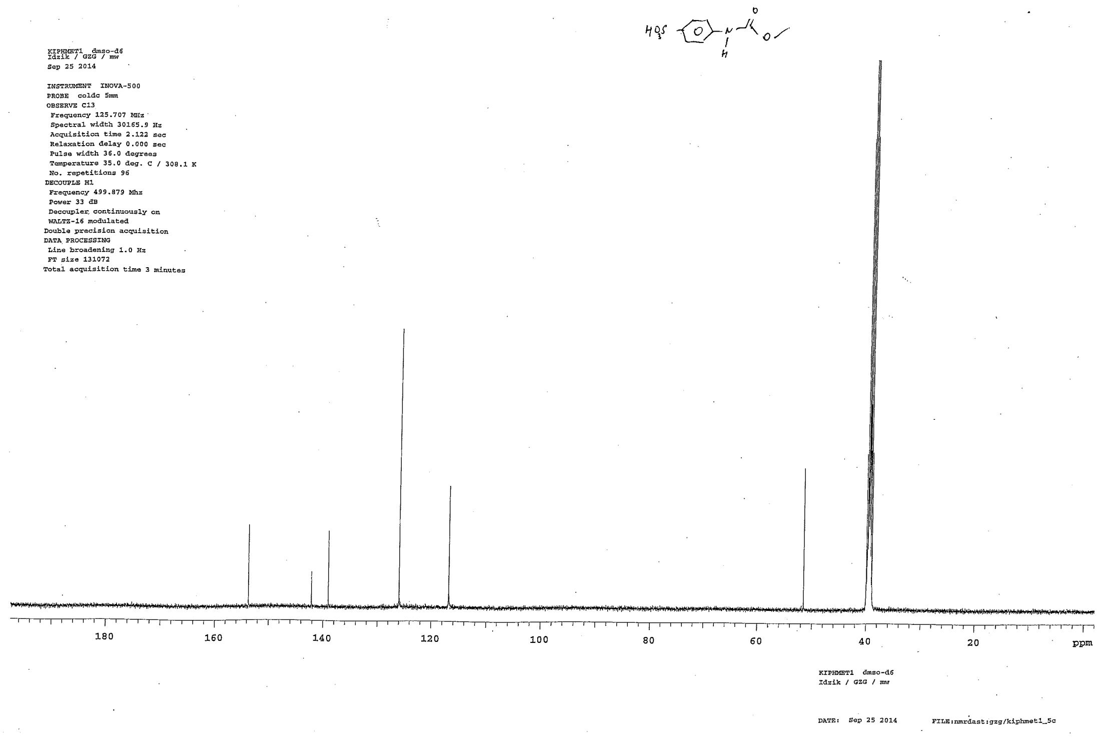

Figure S8. ^{13}C-NMR spectrum of 4-[(methoxycarbonyl)amino]benzenesulfonic acid (**4**). ^{13}C-NMR (300 MHz, DMSO-d_6) δ, ppm, 153.70, 142.13, 139.05, 125.99, 116.84, 51.53.

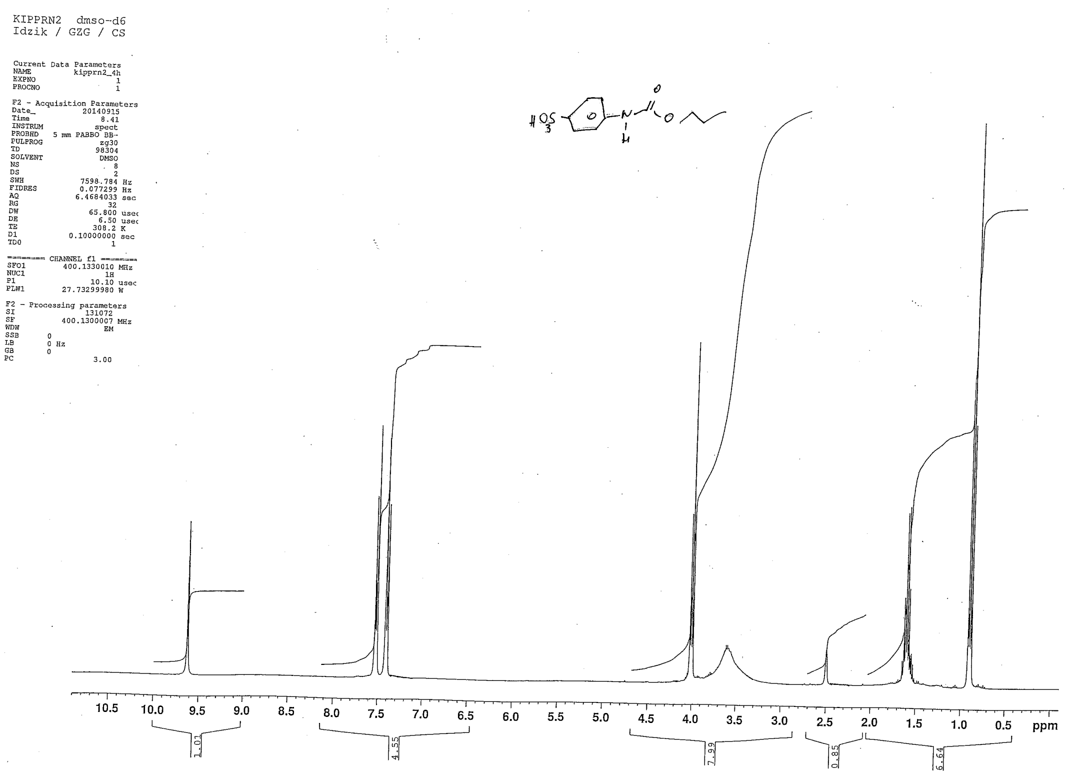

Figure S9. ^{1}H-NMR spectrum of 4-[(propoxycarbonyl)amino]benzenesulfonic acid (**5**). ^{1}H-NMR (300 MHz, DMSO-d_6) δ, ppm, 9.63 (s, 1H), 7.52 (d, J = 8.8 Hz, 2H), 7.41 (d, J = 8.4 Hz, 2H), 4.01 (t, J = 6.6 Hz, 2H), 1.65–1.56 (m, 2H), 0.90 (t, J = 7.4 Hz, 3H).

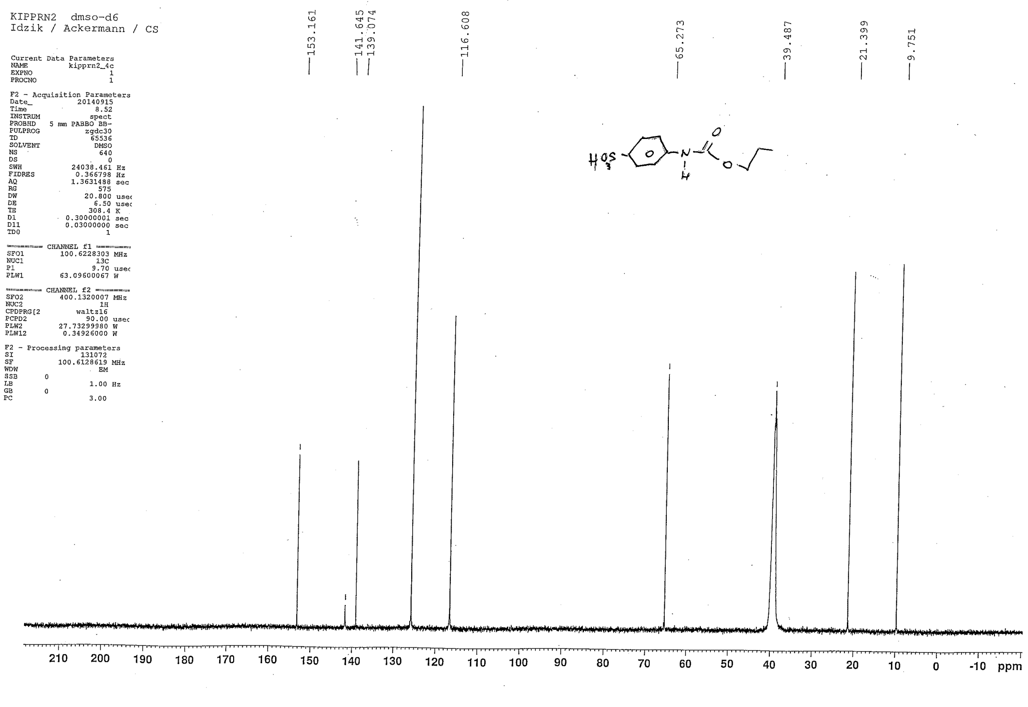

Figure S10. ^{13}C-NMR spectrum of 4-[(propoxycarbonyl)amino]benzenesulfonic acid (**5**). ^{13}C-NMR (300 MHz, DMSO-d_6) δ, ppm, 153.17, 141.66, 139.08, 125.76, 116.62, 65.29, 21.42, 9.77.

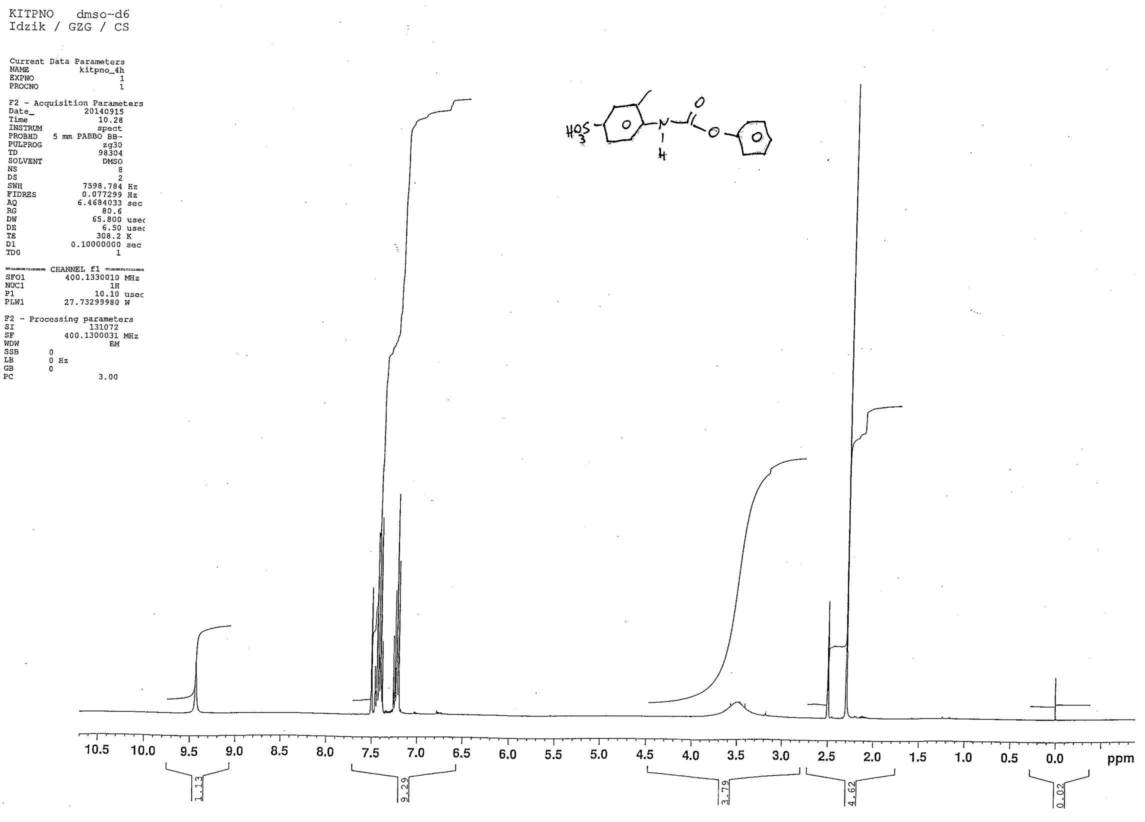

Figure S11. ^{1}H-NMR spectrum of 3-methyl-4-[(phenoxycarbonyl)amino]benzenesulfonic acid (**6**). ^{1}H-NMR (300 MHz, DMSO-*d*$_6$) δ, ppm, 9.41 (s, 1H), 7.47 (s, 1H), 7.42–7.35 (m, 4H), 7.23 (t, J = 6.0 Hz, 1H), 7.20 (d, J = 6.0 Hz, 2H), 2.29 (s, 3H).

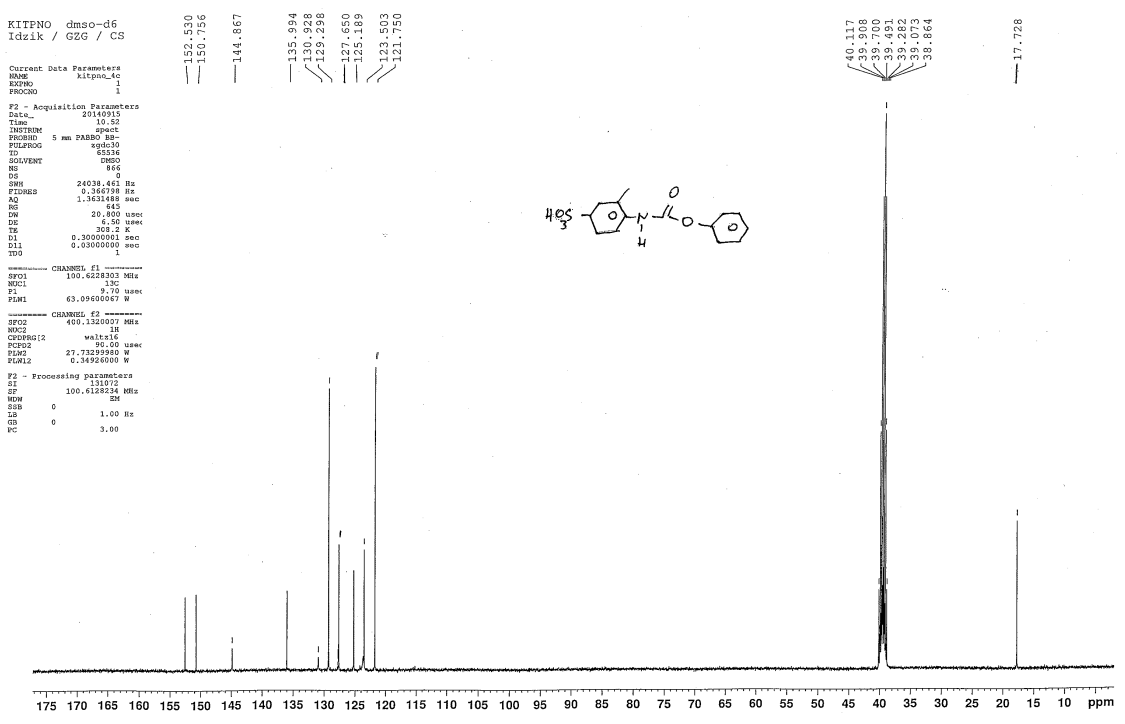

Figure S12. ^{13}C-NMR spectrum of 3-methyl-4-[(phenoxycarbonyl)amino]benzenesulfonic acid (**6**). ^{13}C-NMR (300 MHz, DMSO-*d*$_6$) δ, ppm, 152.53, 150.76, 144.87, 135.99, 130.93, 129.30, 127.65, 125.19, 124.20, 123.50, 121.75, 17.73.

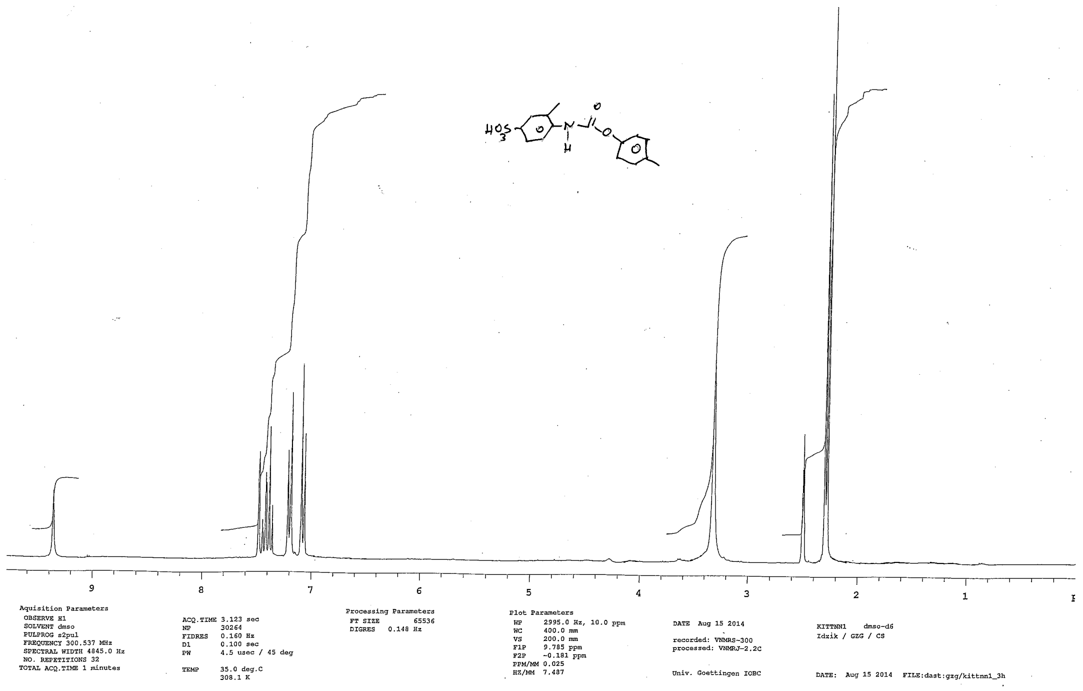

Figure S13. ^{1}H-NMR spectrum of 3-methyl-4-{[(4-methylphenoxy)carbonyl]amino} benzenesulfonic acid (**7**). ^{1}H-NMR (300 MHz, DMSO-d_6) δ, ppm, 9.37 (s, 1H), 7.48 (s, 1H), 7.43 (d, J = 8.4 Hz, 1H), 7.37 (d, J = 8.4 Hz, 1H), 7.21 (d, J = 8.4 Hz, 2H), 7.08 (d, J = 8.4 Hz, 2H), 2.31 (s, 3H), 2.28 (s, 3H).

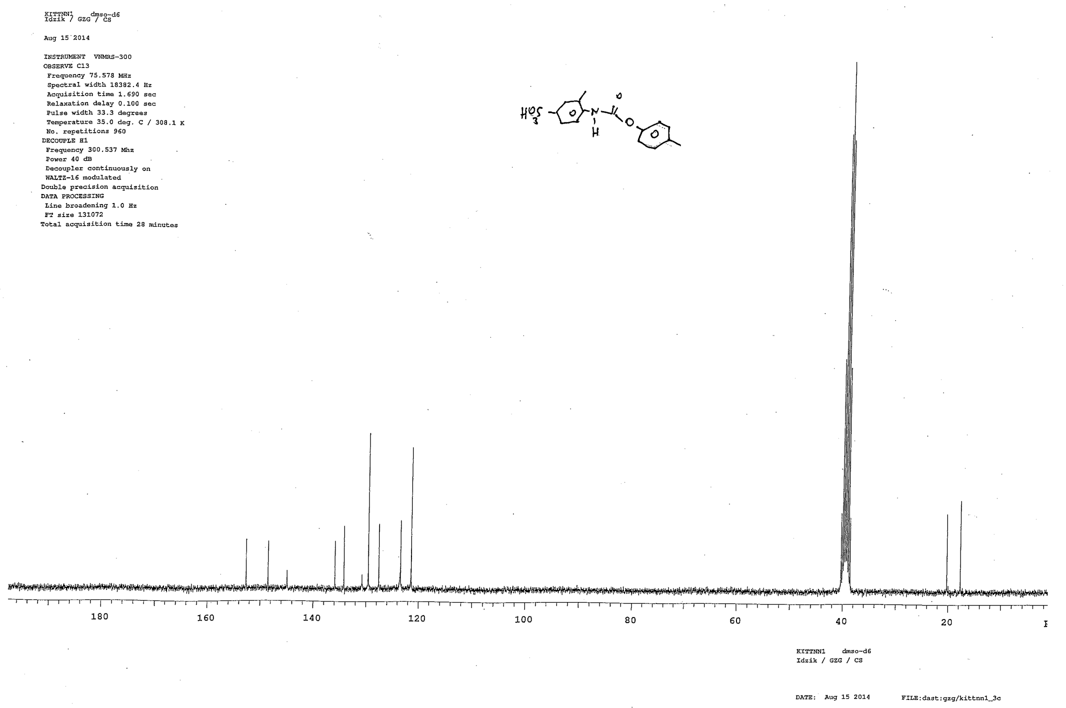

Figure S14. ^{13}C-NMR spectrum of 3-methyl-4-{[(4-methylphenoxy)carbonyl]amino} benzenesulfonic acid (**7**). ^{13}C-NMR (300 MHz, DMSO-d_6) δ, ppm, 152.62, 148.50, 144.96, 135.92, 134.24, 130.76, 129.58, 127.58, 123.56, 123.43, 121.43, 20.26, 17.69.

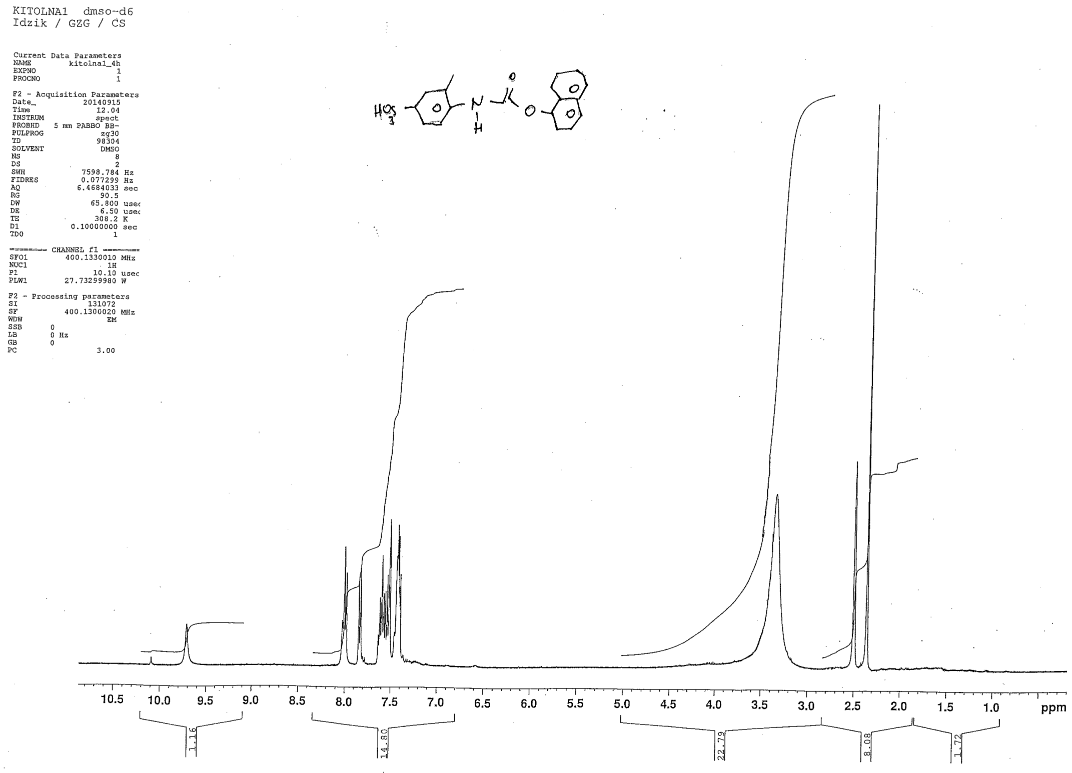

Figure S15. ^{1}H-NMR spectrum of 3-methyl-4-{[(naphthalen-1-yloxy)carbonyl]amino} benzenesulfonic acid (**8**). ^{1}H-NMR (300 MHz, DMSO-d_6) δ, ppm, 9.72 (s, 1H), 8.03–7.98 (m, 2H), 7.84 (d, J = 8.0 Hz, 1H), 7.64–7.52 (m, 4H), 7.46–7.40 (m, 3H), 2.36 (s, 3H).

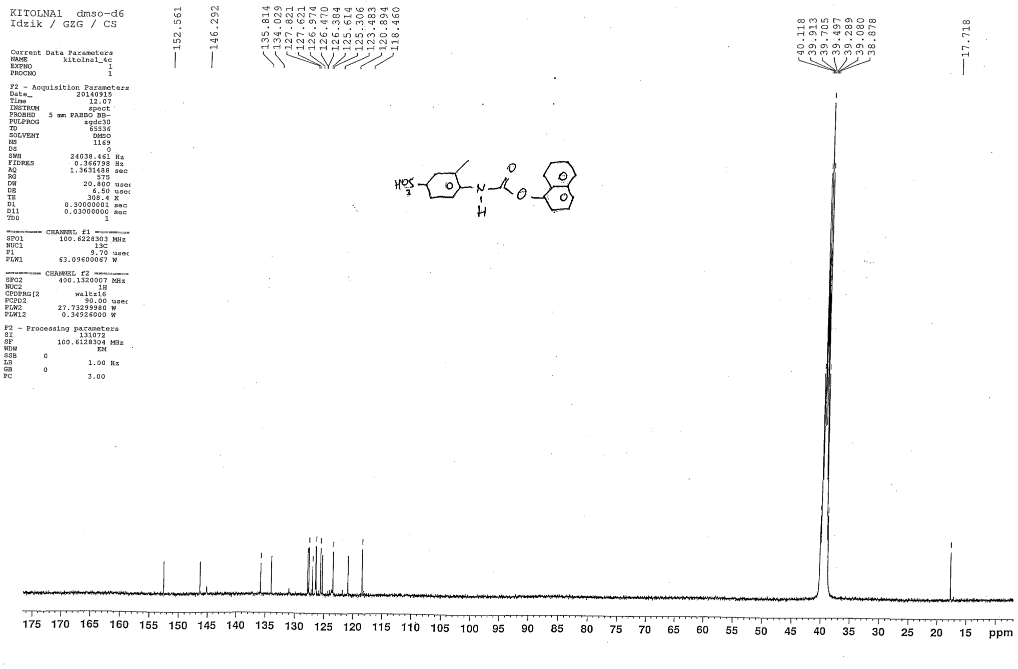

Figure S16. ^{13}C-NMR spectrum of 3-methyl-4-{[(naphthalen-1-yloxy)carbonyl]amino} benzenesulfonic acid (**8**). ^{13}C-NMR (300 MHz, DMSO-d_6) δ, ppm, 152.57, 146.30, 145.14, 135.83, 134.04, 130.99, 127.83, 127.63, 126.99, 126.48, 126.40, 125.63, 125.32, 124.07, 123.50, 120.91, 118.47, 17.74.

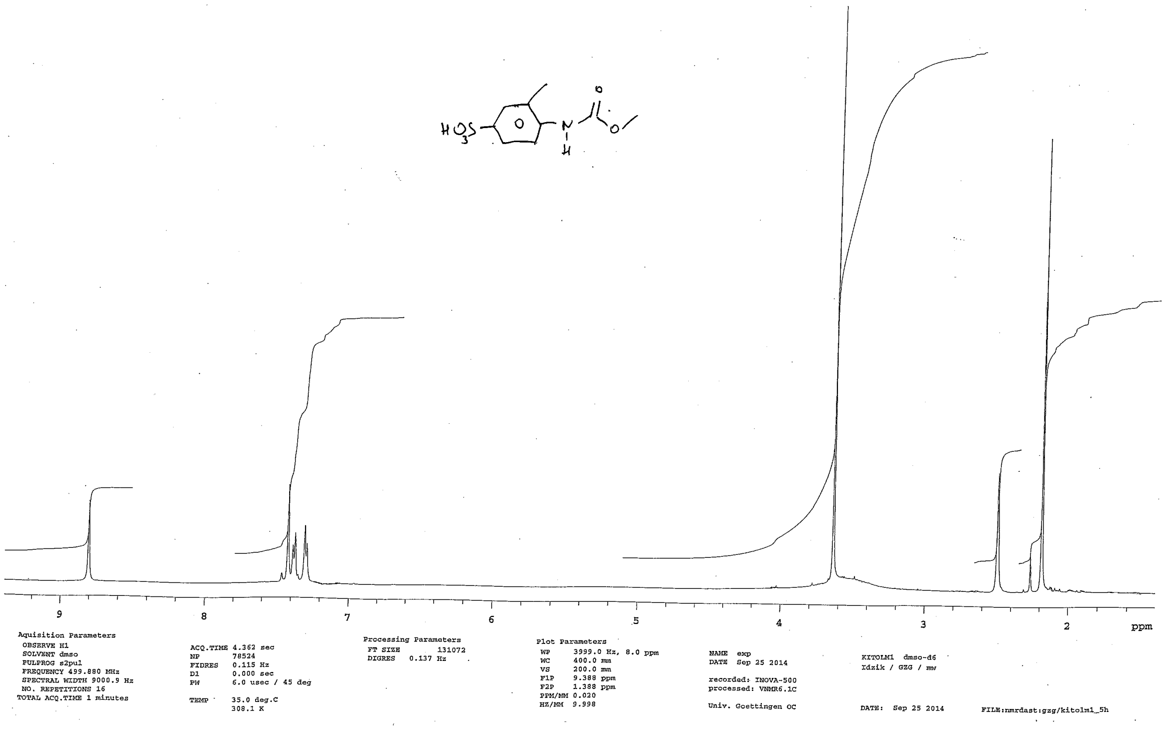

Figure S17. ^{1}H-NMR spectrum of 4-[(methoxycarbonyl)amino]-3-methylbenzenesulfonic acid (**9**). ^{1}H-NMR (300 MHz, DMSO-d_6) δ, ppm, 8.80 (s, 1H), 7.42 (s, 1H), 7.38 (d, J = 7.5 Hz, 1H), 7.30 (d, J = 7.5 Hz, 1H), 3.64 (s, 3H), 2.19 (s, 3H).

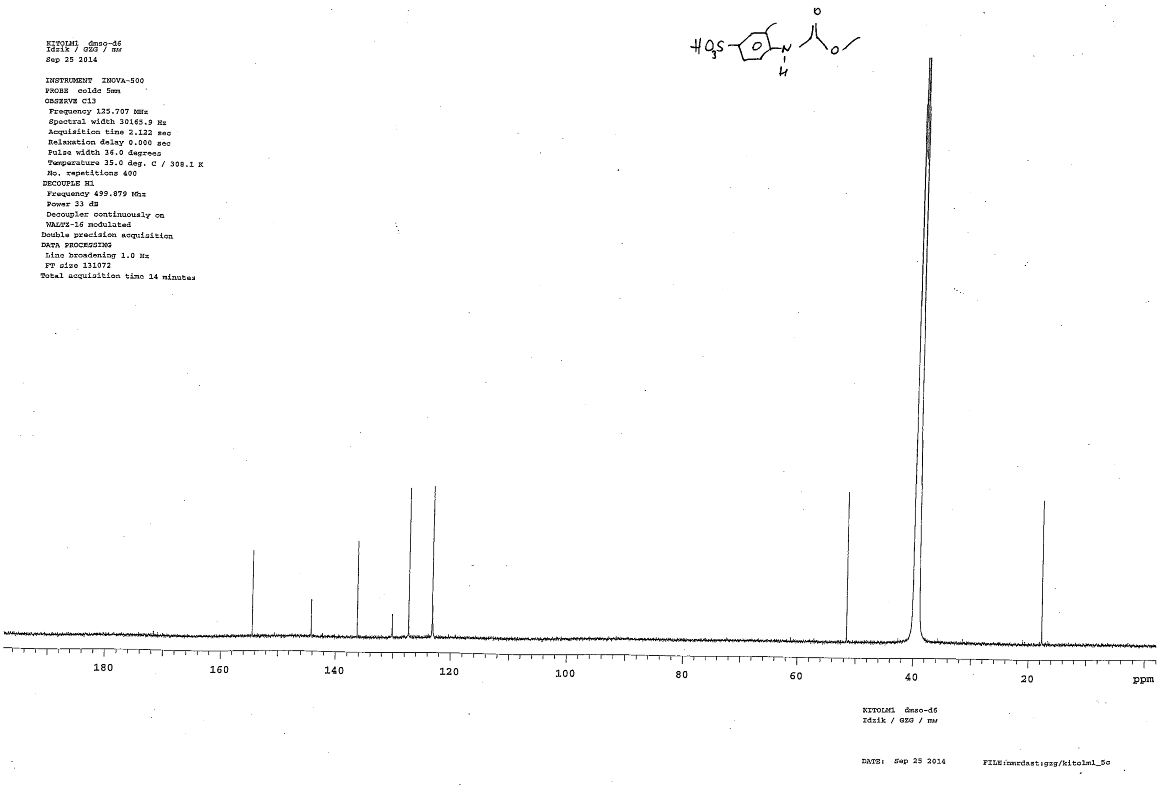

Figure S18. ^{13}C-NMR spectrum of 4-[(methoxycarbonyl)amino]-3-methylbenzenesulfonic acid (**9**). ^{13}C-NMR (300 MHz, DMSO-d_6) δ, ppm, 154.47, 144.23, 136.26, 130.17, 127.31, 123.20, 123.10, 51.56, 17.68.

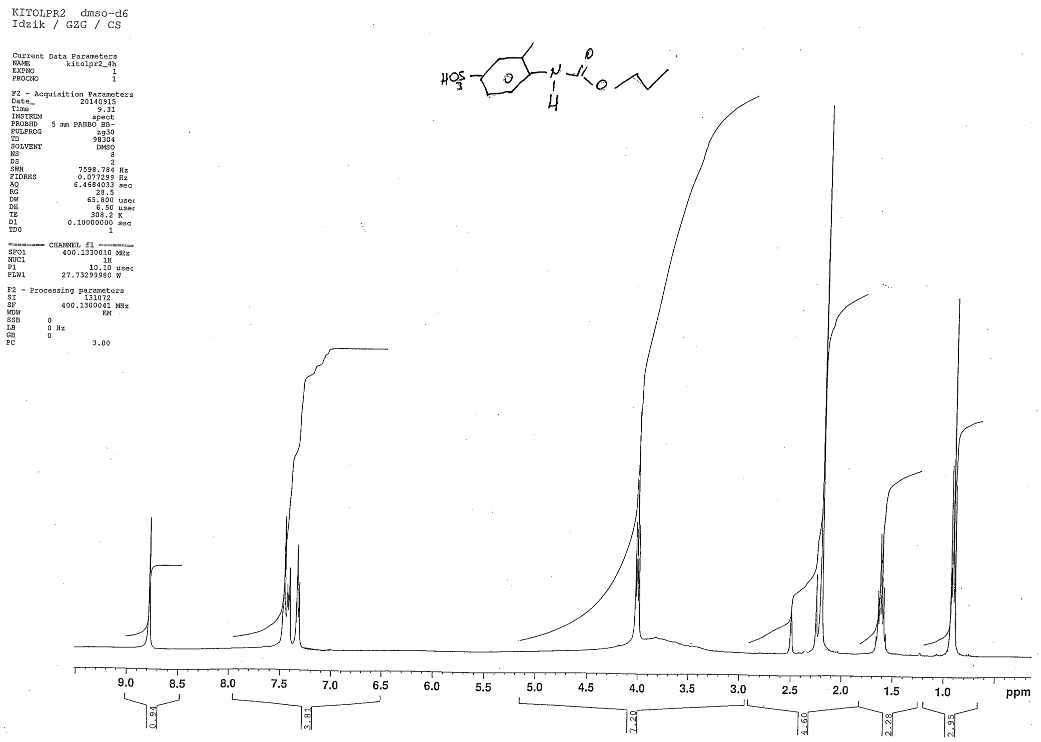

Figure S19. ^{1}H-NMR spectrum of 3-methyl-4-[(propoxycarbonyl)amino]benzenesulfonic acid (**10**). ^{1}H-NMR (300 MHz, DMSO-d_6) δ, ppm, 8.78 (s, 1H), 7.46 (s, 1H), 7.42 (d, J = 8.4 Hz, 1H), 7.32 (d, J = 8.4 Hz, 1H), 4.01 (t, J = 6.6 Hz, 2H), 2.20 (s, 3H), 1.66–1.57 (m, 2H), 0.91 (t, J = 7.4 Hz, 3H).

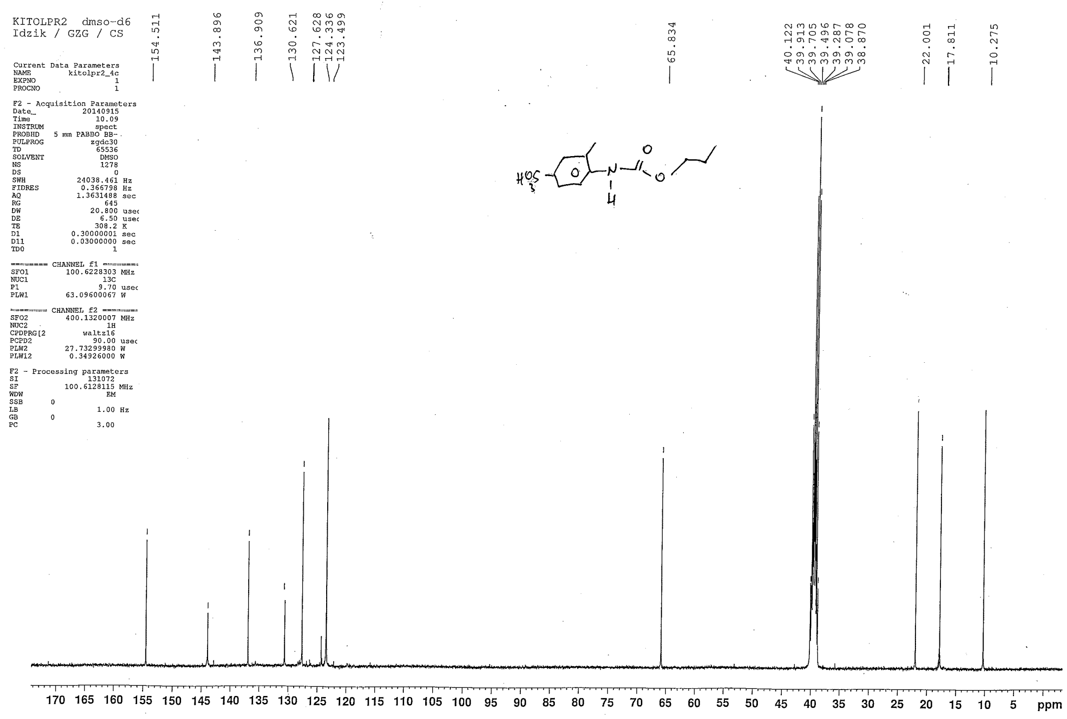

Figure S20. ^{13}C-NMR spectrum of 3-methyl-4-[(propoxycarbonyl)amino]benzenesulfonic acid (**10**). ^{13}C-NMR (300 MHz, DMSO-d_6) δ, ppm, 154.52, 143.90, 136.92, 130.63, 127.64, 124.35, 123.51, 65.85, 22.02, 17.83, 10.30.
